# Comparative characterization of the reassortant Orthobunyavirus Ngari with putative parental viruses, Bunyamwera and Batai: *in vitro* characterization and *ex vivo* stability

**DOI:** 10.1099/jgv.0.001523

**Published:** 2020-12-01

**Authors:** M. Fausta Dutuze, E. Handly Mayton, Joshua D. Macaluso, Rebecca C. Christofferson

**Affiliations:** ^1^​ Department of Pathobiological Sciences, School of Veterinary Medicine, Louisiana State University, Baton Rouge, LA 70803, USA; ^2^​ Rwanda Institute of Conservation and Agriculture, Gashora, Bugesera, Rwanda

**Keywords:** Orthobunyavirus, Bunyavirus, viral stability, Bunyamwera, Batai, Ngari

## Abstract

Bunyamwera (BUNV), Batai (BATV) and Ngari (NRIV) are mosquito-borne viruses that are members of the genus *Orthobunyavirus* in the order Bunyavirales. These three viruses are enveloped with single-stranded, negative-sense RNA genomes consiting of three segments, denoted as Small (S), Medium (M) and Large (L). Ngari is thought to be the natural reassortant progeny of Bunyamwera and Batai viruses. The relationship between these ‘parental’ viruses and the ‘progeny’ poses an interesting question, especially given that there is overlap in their respective transmission ecologies, but differences in their infection host ranges and pathogenesis. We compared the *in vivo* kinetics of these three viruses in a common laboratory system and found no significant difference in growth kinetics. There was, however, a tendency of BATV to have smaller plaques than either BUNV or NRIV. Furthermore, we determined that all three viruses are stable in extracellular conditions and retain infectivity for a week in non-cellular media, which has public health and biosafety implications. The study of this understudied group of viruses addresses a need for basic characterization of viruses that have not yet reached epidemic transmission intensity, but that have the potential due to their infectivity to both human and animal hosts. These results lay the groundwork for future studies of these neglected viruses of potential public and One Health importance.

## Introduction

Bunyamwera (BUNV), Batai (BATV) and Ngari (NRIV) are mosquito-borne viruses that are members of the genus *Orthobunyavirus* in the family Bunyaviridae [1–3]. These three viruses are enveloped with single-stranded, negative-sense RNA genomes consisting of three segments, denoted as Small (S), Medium (M) and Large (L). The S segment encodes the nucleocapsid, the M segment encodes envelope glycoproteins and the L segment encodes polymerase protein [3–6]. NRIV is the natural reassortant of BUNV and BATV and results from a combination of BUNV S and L segments and BATV M segment [1, 3, 7]. The relationship between these ‘parental’ viruses and the ‘progeny’ poses an interesting question, especially given that there is overlap in their respective transmission ecologies, but differences in their infection host ranges and pathogenesis [8].

BUNV, the prototype of the genus *Orthobunyavirus*, is the most characterized of the three, followed by BATV and lastly NRIV [1, 8–11]. BUNV and NRIV have been identified in several African countries between 1943 and 2012 [8, 12–19]. BATV was first identified in Uganda in 1967, was highly suspected in Sudan based on serological analysis in 1988, and otherwise has not been detected in Africa since, although it has been isolated in Europe and Asia in the past decade [8, 12–20]. There have been no molecularly confirmed detections of BUNV and BATV concomitantly, although NRIV itself is evidence that this event must have occurred.

Investigations of NRIV have mostly focused on the genomic (dis)similarities with other members of the family Bunyaviridae and the similarity of its clinical manifestations with Rift Valley Fever virus (RVFV), an important bunyavirus of the genus *Phlebovirus* [1, 3, 4, 21, 22]. All three of these Orthobunyaviruses cause disease in domestic ruminants and humans, with NRIV being associated with haemorrhagic fever in humans [1, 3, 4, 7]. Despite the potential for one or all these viruses to present an emergent public health threat, they remain relatively understudied and a comparative understanding of the biology of BUNV, BATV and NRIV has not been undertaken. Here we present a comparison of these three related Orthobunyaviruses in common laboratory cell culture systems, compare growth kinetics and plaque morphologies, and determine their stability in extracellular conditions.

## Methods

### Viruses and comparative plaque phenotype

6547–8, MM2222 and DAK-AR D28542 are the strain designations of BUNV, BATV and NRIV respectively used throughout this study. 6547–8 is the prototype strain of BUNV isolated from *Aedes* mosquitoes in Uganda in 1943 [23]. MM2222 is the prototype of BATV isolated from *Culex* mosquitoes in Malaysia in 1955 [8]. The DAK-AR D28542 strain of NRIV was isolated from *Aedes* mosquitoes in Senegal in 1985 [18]. The viruses were obtained from the World Reference Center for Emerging Viruses and Arboviruses at the University of Texas Medical Branch. All viruses were provided in lyophilized form and were reconstituted by adding 1 ml of cell culture media to the vials. The passage histories at the time they were received were as follows: Suckling mouse brain (SM)47/Vero (V)2 for BUNV; SM3/V2 for BATV; and SM4/baby hamster kidney (BHK)1/V2 for NRIV. After reconstitution, the viruses were passaged once more on Vero African green monkey kidney cells (ATCCs) and stocks were collected after observation of the onset of cytopathic effects (CPEs) at 2 days post-inoculation (dpi), 5 dpi and 3 dpi for BUNV, BATV and NRIV, respectively by sampling 100 µl of supernatant. The resulting stocks were used for all studies herein. Viral titres were determined by standard plaque assay techniques as reported previously [24].

Briefly, six-well plates were seeded with Vero cells and grown until approximately 85 % confluency. The first overlay was performed by inoculating each well with 100 µl of a virus of one of the 1 : 10 serial dilutions prepared in sterile cell culture media. Plates were rocked for 15 min to maximize contact with the monolayer. The first overlay – SeaPlaque agar-based – was then applied to each well at a volume of 3 ml per well. Plates were incubated under 5 % CO_2_ at 37 °C for 3 days (BUNV and NRIV) or 4 days (BATV). The second overlay containing Neutral Red Stain was then applied at a volume of 1.5 ml into each well and plates were again incubated overnight. Plates were then read at 4 dpi for BUNV and NRIV and 5 dpi for BATV. The calculated titres were 6.65×10^6^ p.f.u. ml^−1^ for BUNV, 2.6×10^7^ p.f.u. ml^−1^ for BATV and 5.7×10^7^ p.f.u. ml^−1^ for NRIV.

Plaques were phenotypically compared following a plaque assay. We took pictures of plaque assay wells with comparative numbers of plaques (NRIV 10^−5^ dilution, BATV 10^−4^ dilution, BUNV 10^−4^ dilution). Pictures were then analysed using ImageJ software (version 1.53c, https://imagej.nih.gov/ij/index.html). Each picture included a ruler and a line was drawn to correspond to 1 cm and converted to the number of pixels (Figs S1–S3, available in the online version of this article). Plaques were traced using the polygon tool by a single individual to avoid user bias and variability, and the area calculated by the program. Thirty randomly chosen plaques were measured of BATV, 25 of BUNV and 28 for NRIV to achieve a sufficient sample size for plaque area. Exclusion of other plaques in the well was by chance, not based on exclusion criteria. Visual inspection of the plaques and measurement was subsequently performed by the same individual for descriptive determination of potential differences in morphology to reduce variability.

### Viral RNA, qRT-PCR assay design

RNA was extracted using the Kingfisher (Thermo-Fisher) automated extraction platform according to the manufacturer’s instructions as reported previously [25]. Quantitative reverse transcriptase PCR (qRT-PCR) primers and probes targeting conserved regions of the M and L segments of BUNV, BATV and NRIV were designed using the PrimerQuest tool (www.idt.dna). Primer and probe sequences and GenBank accession numbers on which primers were based are given in Table 1 [26–31]. Prior to use in the experiments, primer–probe sets were tested on viral stocks for sensitivity and cross-reactivity, and standard curves generated were informed by the plaque assays used to quantify the viruses. Nucleic acid amplification was performed by qRT-PCR using the SuperScript III One-Step RT-PCR System with Platinum Taq DNA Polymerase (Invitrogen, Cat. No. 1.1732–088) as previously described [32]. Plates were centrifuged for 2 min at 2000 r.p.m. and then loaded into the Roche Lightcycler 480 II. The following thermal profile was used: one cycle of reverse transcription for 10 min at 50 °C, 15 min at 95 °C for reverse transcriptase inactivation and DNA polymerase activation followed by 40 amplification cycles of 15 s at 95 °C (annealing-extension step) and 30 s at 40 °C (cooling) as previously reported [25, 33]. Results from qRT-PCR are reported as genome equivalents ml^–1^.

**Table 1.** Sequences of primers and probes used for qRT-PCR

Virus and segment targeted	Primer/probe	Sequence	GenBank accession no
BUNV- M	Forward primer	5′-GCTTATGGATGGGCGTACAA-3′	M11852
	Reverse primer	5′-GGAGCCACAGACACAATATGA-3′	
	Probe	5′-/5Cy5/ATGCACTTGCGGATTGGCAT/3lAbRQSp/-3′	
BUNV-L	Forward primer	5′-GCCACTTTGCTGATTCCTTTG-3′	X14383
	Reverse primer	5′-CTAACCTTGTAGTGCTGGCTAATA-3′	
	Probe	5′-/56-FAM/TGGAAGAGG/ZEN/CAAGCAGATTGAGCT/3lABkFQ/-3′	
BATV-M	Forward primer	5′-GCATGTGGAAACTCACCAAATTA-3′	JX846596.1
	Reverse primer	5′-ATTCTTGTGAGGCAGGGATTAG-3′	
	Probe	5′-/5Cy5/AAGGGAGAAGTGTGGTGTTCAGGT/3BHQ_2/-3′	
BATV-L	Forward primer	5′-CACTCTACCAGCTGCATTCTAC-3′	JX846597.1
	Reverse primer	5′-GTTGACCACGGTTCACTACTT-3′	
	Probe	5′-/56-FAM/ACAGCTGCA/ZEN/GGG ATAATTAACTGGACC/3lABkFQ/-3′	
NRIV-M	Forward primer	5′-TATAGGCCCTTTACAGCAAGTG-3′	KC608153
	Reverse primer	5′-GCTGCATCCAGGTCTGATATT-3′	
	Probe	5′-/5Cy5/ACATGCGACGATAAAGCAAGCAGA/3lAbRQSp/−3’	
NRIV-L	Forward primer	5′-GCGAAACCGTGTAGAAAGTAGA-3′	KC608152
	Reverse primer	5′-CCCTGAAATCACCGACCTTTAT-3′	
	Probe	5′-/56-FAM/AGCTTGTGA/ZEN/AAGTGCTTATTGTTGTGATGC/3lABkRQ/-3′	

### *In vitro* growth kinetics under standard growth conditions

To compare the growth kinetics of these viruses in a commonly used cell culture system, 85–90 % confluent six-well plates of Vero cells grown in optimal medium (88 % Medium 199 with Earle’s Salts, 10 % FBS and 2 % antibiotic-antimycotic) – hereafter simply referred to as ‘cell culture media’ – were infected with serially diluted BUNV, BATV and NRIV from 10^6^ to 10^1^ p.f.u. ml^−1^ in cell culture media. Prior to infection, medium was removed from each well and 100 µl of each dilution for each of the three viruses was inoculated onto individual wells. The wells were then rocked at room temperature for 30 min and then incubated for 30 min at 37 °C with 5 % CO_2_. After incubation, 2 ml of new optimal medium was added to each well and stored at 37 °C with 5 % CO_2_. Negative control wells with uninoculated cell culture media were prepared following the same protocol. In total, 100 µl of supernatant was collected from each well at 1–7 dpi and stored at −80 °C until RNA extraction and qRT-PCR could be performed. These comparisons were run in triplicate.

In addition, a gradual CPE was observed under an inverted microscope at 20× at various days post-inoculation and pictured with the DINO capture function. Different qualitative scores were given to each well at 1–12, 14 and 30 dpi according to the relative proportion of dead cells compared to healthy cells. Cells were scored as (0) no observed CPE, (1) a few dead cells, (2) approximately one-third of cells are dead, (3) approximately half of cells are dead, (4) approximately two-thirds of cells are dead, (5) very few cells still attached (6) and all cells are dead (no cells attached). This was done by a single observer to avoid introducing variability and/or bias.

### Stability in extracellular and cell-free media

Long-term infectivity of the virus under extracellular conditions (when all cells were dead) was investigated after noting recovery of high levels of viral RNA at 30 dpi. This was done by inoculating 100 µl of the mixture collected at 30 dpi from wells previously inoculated with 10^6^, 10^4^ and 10^1^ p.f.u. ml^−1^ of each of the three viruses onto 85–90 % confluent six-well plates of Vero cells. To determine infectiousness of the virus, we collected supernatant at 1, 3, 5 and 7 dpi and replication was determined by observing a positive growth curve when tested for viral RNA via qRT-PCR.

We further investigated the potential stability of these viruses in cell-free conditions. To do this, 100 µl of the stock of each of three viruses was put into 2 ml of cell-free M199 2× media in 2 ml Eppendorf tubes. The sealed tubes were incubated at human body temperature (37 °C) and 100 µl of the mixture was collected from each tube at 1, 3, 5, 7, 14 and 30 dpi and stored at −80 °C until further processing. To investigate stability, we then collected 100 µl of the mixture at 30 dpi and inoculated it onto confluent Vero cells and tested for positive growth as described above. These experiments were run in triplicate.

### Comparative inactivation by Triton-X-100

We investigated comparative inactivation of these viruses by Triton-X-100 as this detergent has been used to inactivate other enveloped viruses such as Ebola virus [34] and influenza [35]. For this, 1 % Triton-X-100 was added to the stock of each of the three viruses (5 µl of Triton-X-100 in 495 µl viral stock) and the mix was incubated at room temperature for 1 h and stored at −80 °C until further use. To test the efficacy of Triton-X-100 inactivation capacity on these viruses, the viral infectivity of the supposedly inactivated viruses was investigated on Vero cells. Prior to infections, Triton-X-100 was removed using the column-based absorption Detergent OUT kit according to the manufacturer's instructions (Millipore Sigma, Cat. No. 2114). We used 85–90 % confluent six-well plates of Vero cells. After removal of the 2 ml cell culture media, 100 µl of each virus was inoculated into a well. Plates were then rocked for 30 min and incubated at 37 °C with 5 % CO_2_ for 30 min before adding 2 ml of new cell culture media. The presence of CPEs was observed and 100 µl of the supernatant was collected every day until 7 dpi. RNA extraction and qRT-PCR were performed on collected supernatants. The experiments were also run in triplicate.

### Statistics

To determine differences in plaque size, the area of plaques was compared among viruses by a Kruskal–Wallis non-parametric *t*-test, after determining the data were not normally distributed (Shapiro Wilk, *P*<0.05). For pairwise comparisons, the Wilcoxon rank-sum test with a Bonferroni correction for multiple comparisons was used.

The R package *Growthcurver* was used to analyse growth kinetics of BUNV, BATV and NRIV in intracellular medium under standard conditions as well as in extracellular medium [36]. *Growthcurver* determines the area under the curve (AUC) and doubling time (DT) used to characterize growth curves. AUC is determined under the logistic curve in the form of two metrics (the logistic AUC or AUC-L) that integrate different parameters such as the population size at the beginning of the growth curve, maximum possible population in a particular environment, and intrinsic growth of the population. DT is the time that a population takes for the number of individuals to double. *Growthcurver* determines the fastest DT which occurs when the population is maximizing its growth potential [37, 38]. For all growth conditions and for each inoculation dose of each virus, *Growthcurver* analysis was performed for all replicates individually and ANOVA on AUC-L values was used to establish comparison between viruses and inoculation doses among different growth cell conditions. In case of significance, Tukey's *post-hoc* test was performed for pairwise comparisons. ANOVA was performed to compare the DT of the curves between cell growth conditions and inoculation doses among viruses.

## Results

### Plaque assay and plaque morphology

**Fig. 1.**
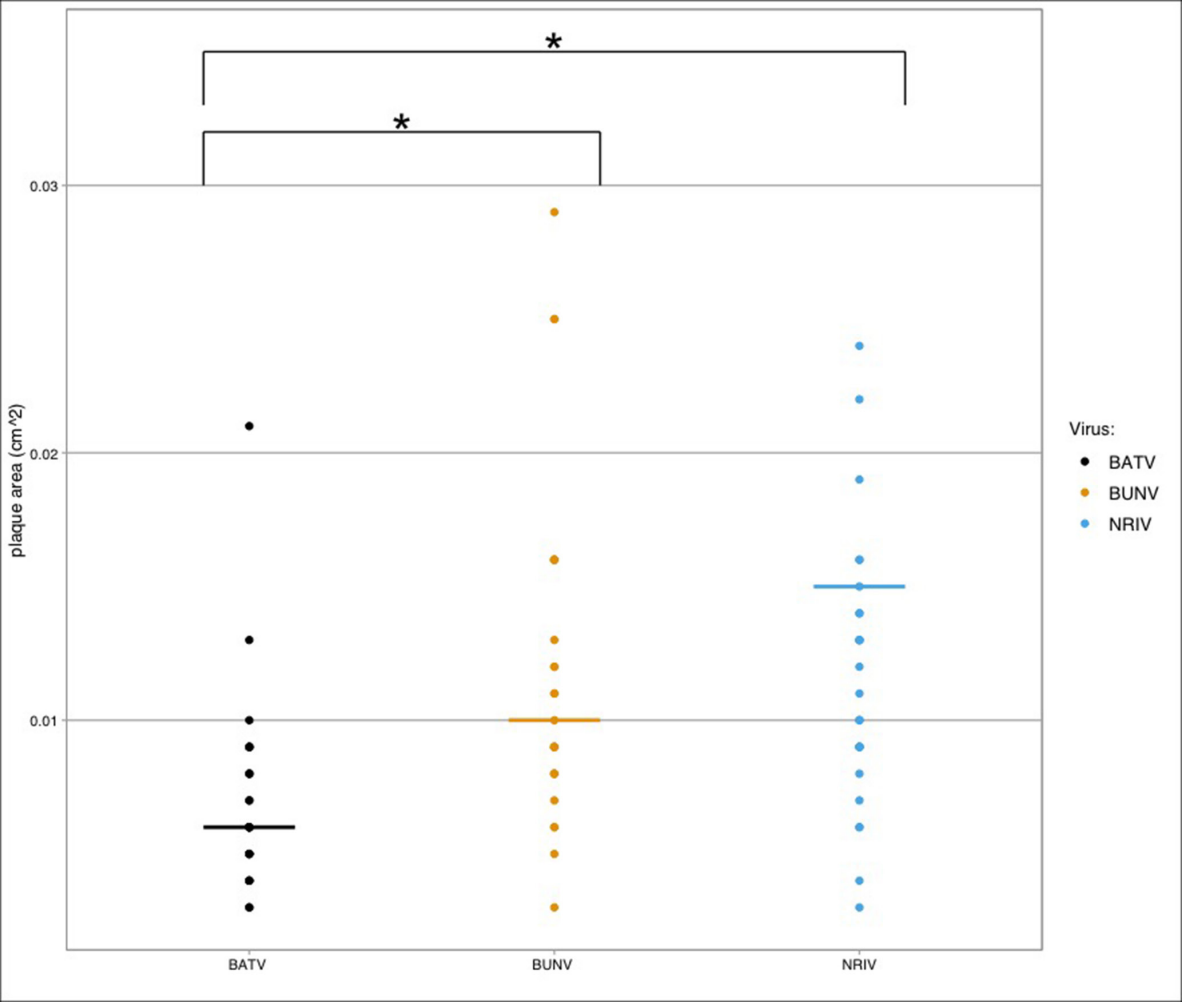
Comparisons of plaque area (cm^2^) among the three viruses show that BATV had smaller overall plaque area (lines represent the median). *Statistically significant differences in plaque size trends when pairwise comparisons were done via Wilcoxon's rank-sum test.

The median plaque area of BATV was 0.006 cm^2^ (range: 0.003–0.021 cm^2^), the median area of BUNV plaques was 0.01 cm^2^ (range: 0.003–0.029 cm^2^) and the median area of NRIV plaques was 0.0105 cm^2^ (range: 0.003–0.024 cm^2^). Despite similarity in their overall range, BATV produced plaques that had a tendency to be smaller overall than either BUNV or NRIV (*P*<0.05 by Wilcoxon's rank-sum test), while BUNV and NRIV plaques did not have significantly different trends in plaque size (Fig. 1). Aside from size, plaques did not appear to have vastly different morphology, each having both small and larger plaques (Figs S1–S3).

### Sensitivity and cross-reactivity of qRT-PCR

We decided to design and test a combination of the M and L primers to differentiate among the viruses. This is because, in order to differentiate viral identity when not in mixed culture, only two of the three segments are necessary. The designed qRT-PCR primers for each virus successfully amplified appropriate self-segments. In addition, there was a successful cross-amplification between the BUNV and NRIV L segments. However, the cross-amplification between BATV and NRIV M segments was not consistent: the BATV M primers cross-amplified the NRIV M segment, but the NRIV M primers did not cross-amplify the BATV M segment (Table S1). For our experimental studies, because no co-infection was performed, only homologous primers were used for the detection of viral RNA of each virus. For all three viruses, the sensitivity of both L and M primers was as low as 10^1^ genome equivalents ml^–1^.

### Comparison of growth curves and viral stability

We investigated the progression of CPEs by observing wells inoculated with each virus at each inoculation dose. At the higher (>10^3^ p.f.u. ml^−1^) doses, significant CPEs were observed starting at 3–4 dpi for all three viruses. By 8–9 dpi for all three viruses, all cells were detached and rounded, indicating non-viable cell culture. The average scores across the three replicates are given in Table 2.

**Table 2.** Gradual CPEs during persistent infections of BUNV, BATV and NRIV in Vero cells

Virus	Initial titre	1 dpi	2 dpi	3 dpi	4 dpi	5 dpi	6 dpi	7 dpi	8 dpi	9 dpi	10 dpi
BATV	1	0.00	0.33	1.00	1.00	1.00	1.33	3.00	4.33	5.67	6
	2	0.00	1.00	1.33	1.33	1.33	2.67	4.00	4.33	5.67	6
	3	0.00	1.00	1.00	2.33	2.33	3.00	4.33	4.33	5.67	6
	4	0.00	1.00	2.00	3.00	3.33	4.33	5.00	6.00	6.00	6
	5	0.00	1.00	2.00	3.67	4.00	5.00	5.33	6.00	6.00	6
	6	0.00	1.33	2.67	4.00	4.33	5.00	5.33	6.00	6.00	6
BUNV	1	0.00	0.33	1.00	2.33	2.33	3.00	4.67	5.00	6.00	6
	2	0.00	0.33	2.00	3.33	3.33	3.67	4.67	5.67	6.00	6
	3	0.00	0.67	2.67	4.33	4.33	4.33	4.67	5.67	6.00	6
	4	1.00	1.00	3.67	5.00	5.00	5.00	5.33	5.67	6.00	6
	5	0.67	1.33	3.33	5.00	5.00	5.00	5.67	5.67	6.00	6
	6	0.67	1.67	3.67	5.00	5.00	5.33	6.00	6.00	6.00	6
NRIV	1	0.00	0.33	1.67	2.00	2.00	2.67	4.33	4.33	5.33	6
	2	0.00	1.00	2.00	3.00	3.00	4.33	5.33	5.33	5.33	6
	3	0.33	1.33	2.00	2.33	2.33	4.33	4.67	5.33	6.00	6
	4	0.33	1.33	2.33	3.33	4.00	4.33	4.33	6.00	6.00	6
	5	0.67	1.67	2.67	4.67	4.67	5.00	5.67	6.00	6.00	6
	6	0.67	1.67	3.00	5.00	5.00	6.00	6.00	6.00	6.00	6

Scale of scores: 0: no CPE observed 1: few dead cells; 2: approximately one-third of cells are dead; 3: approximately half of cells are dead; 4: approximately two-thirds of cells are dead; 5: very few cells still attached; 6: all cells are dead (no cells attached). Values indicate the average from three replicates scored by the same individual to reduce inter-observer variation.

ANOVA of AUC-L from *Growthcurver* showed no significant difference in growth among the three viruses (Fig. S2) nor the interaction term. As expected, a significant difference was found among inoculation doses after Tukey’s post-hoc pairwise comparisons (*P*<0.05) (Table S2, Figs S4 and S5). Notably, there were significant differences among comparisons of: 10^6^–10^1^, 10^6^–10^4^ and 10^4^–10^1^ p.f.u. ml^−1^. This was used to inform the inoculation doses for the stability experiments below. ANOVA and post-hoc comparisons of DTs of infections showed a significant difference between NIRV and BATV viruses, and again DT was largely dose-dependent (Table S3, Fig. S5).

**Fig. 2.**
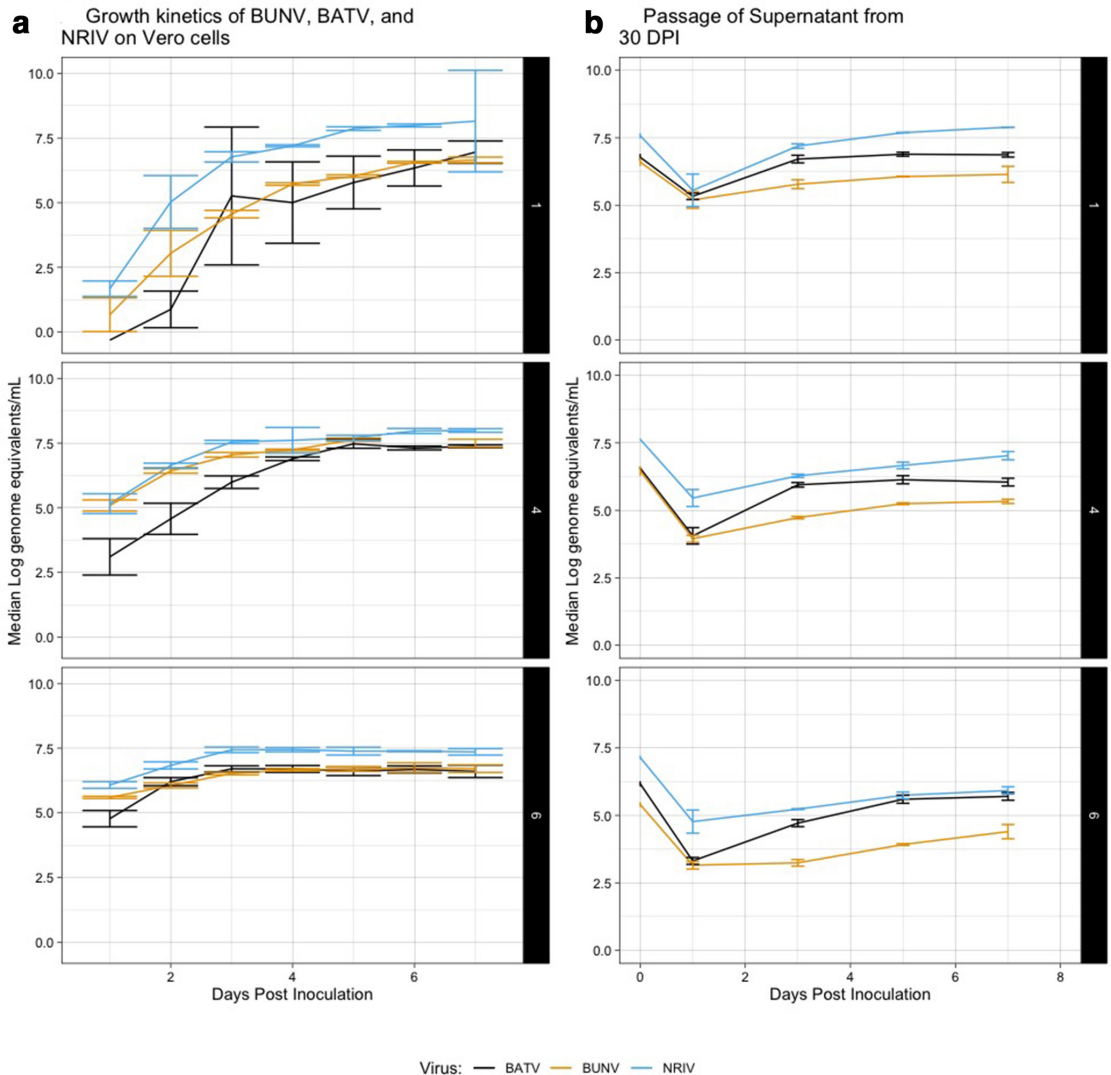
(a) Growth kinetics of the three viruses at different initial inoculation doses of 1, 4 and 6 log_10_ p.f.u. There was no significant difference in growth kinetics among the three viruses in any initial inoculation dose. (b) Growth from supernatant from 30 dpi (at 0 dpi) from the first passage from inoculation doses (ID) 1, 4 and 6 log_10_ p.f.u. over days 1–7 post-inoculation onto fresh cell culture.

Interestingly, relatively high concentrations of viral RNA were detected at 30 dpi when cells were observed to be dead (see Tables 2 and S4, Fig. 2b). We investigated the infectiousness of the viruses at this time point by inoculating supernatant on to fresh Vero cells and monitoring for viral replication via qRT-PCR. This was performed for the following initial inoculation doses: 10^6^, 10^4^ and 10^1^ p.f.u. ml^−1^, which had detectable viral RNA genome equivalents between 5.3 and 7.66 log_10_ ml^−1^ at 30 dpi (Table S4). In all cases, positive growth was detected via qRT-PCR, indicating infectiousness was retained in cell culture after the vast majority of cells were observed to be dead (Fig. 2b).

The supernatant from the lowest first passage dose grew to higher ultimate titres than supernatant from either the 10^4^ or 10^6^ first-pass inoculation doses, despite having similar quantities of viral RNA at 30 dpi. This is probably due to the faster cell death at high initial inoculum compared to lower, resulting in earlier release of viral particles/RNA into the media, which probably resulted in degradation and inactivation of virus. This does suggest that stability is time-sensitive in the presence of dead or dying cells.

**Fig. 3.**
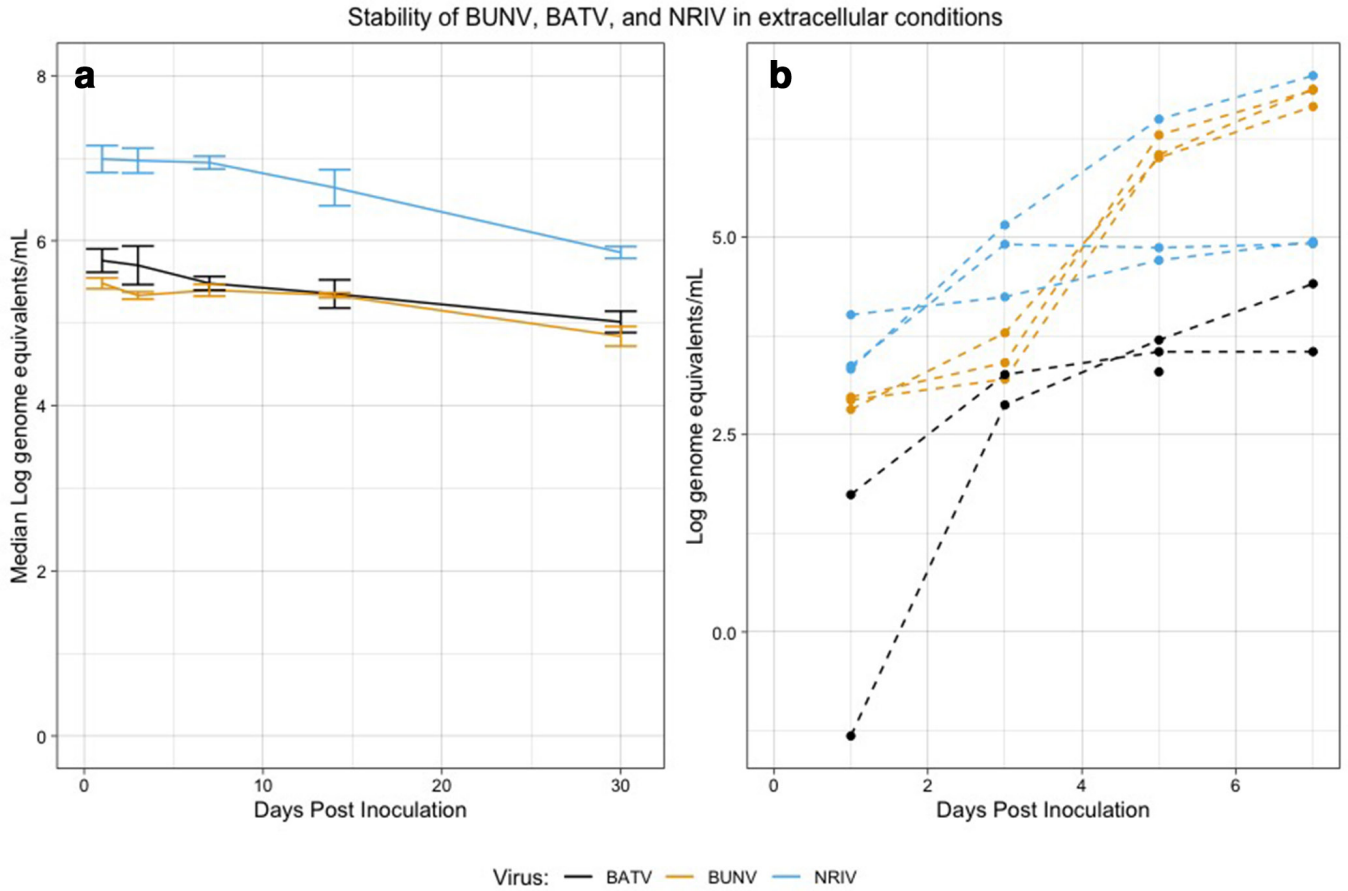
(a) Viral RNA titre at 1, 3, 7, 14 and 30 dpi in acellular media. (b) Growth curves of all three viruses from supernatant collected at 30 dpi from acellular media indicate that all three remained infectious and stable. Error bars are sd across three replicates per virus.

To further investigate the stability of these viruses, we determined the infectivity of viruses kept in cell-free media for 30 dpi. There was a minor reduction in viral RNA detection by qRT-PCR over the 30-day period, but overall, there remained a relatively high concentration of viral RNA at 10^4^–10^5.75^ genome equivalents ml^–1^ (Fig. 3a, Table S5). When we tested these 30-day-old samples for infectivity, we found that in all cases viral replication was observed via qRT-PCR (Fig. 3b). The difference observed between NRIV and the other two viruses is likely to be a consequence of the higher inoculation dose resulting from higher quantities of ‘left-over’ RNA/virus in the 30-day-old sample, as dose dependence was shown in growth kinetics (Fig. 3). Nevertheless, our data indicate stability of these viruses in acellular environments and laboratory conditions.

The range of genome equivalents at 30 dpi onto cell culture was 5.3–6.7 for BUNV, 6.1–6.8 for BATV and 7.1–7.6 for NRIV (Table S5). The ending range of genome equivalents for the cell-free media experiment was 4.7–4.9 for BUNV, 4.8–5.1 for BATV and 5.8–5.9 for NRIV (Table S4). Based on this, we compared the growth curves between fresh cell culture inoculated virus (at an initial inoculum of 6 log p.f.u. ml^−1^), 30-day-old cell culture and cell-free media. The AUC differences among these three origins was not statistically significant (*P*>0.05) by the ANOVA test for BATV and NRIV. BUNV had a significant effect of origin, and post-hoc analysis identified that differences between media-and-fresh and media-and-culture were statistically significant, and the AUC was larger in media (Fig. S6). This is likely to be due to the lower starting titre of the inoculum resulting from degradation during the 30-day incubation.

### Comparative inactivation by Triton-X-100

No CPE was observed on Vero cell monolayers infected with 1 % Triton-X inactivated BUNV, BATV and NRIV at 4 dpi when we had already noted significant CPEs for all three viruses (see Table 1). For all three viruses, viral RNA was detected up to 4 dpi, but when the supernatant was tested for infectivity by inoculation onto fresh Vero cell culture, no growth was observed (Fig. S7). This indicates that Triton-X-100 inactivated all three viruses.

## Discussion

These viruses have the potential to become emergent public health threats to animals and people, so gaining a basic understanding of their kinetics in common laboratory systems is a necessary first step in getting ‘ahead of the curve’ in terms of characterizing these viruses. We observed that BUNV and NRIV produced plaques that were more similar to each other than BATV. This was surprising as the M gene segment – shared between BATV and NRIV – encodes the proteins responsible for cell entry and so it was expected that BATV and NRIV would have more similarity in cell infection patterns. This is an added indication that other factors are mechanistically involved in the infection process, or it may indicate that Vero cells are not the most appropriate cell line for investigating this particular aspect of the infection phenotype [39]. Previous studies on Vero cells identified heterogenous plaque sizes of low-passage BUNV and NRIV isolates, and isolates from different sized plaques were associated with inverse pathogenesis in mice (the large plaque phenotype had reduced virulence in mice compared to the small plaque phenotype) [7]. The authors of that study also suggested that the large plaque phenotype of BUNV replicated more efficiently in Vero cells, but comparisons of growth rates between isolates of each plaque phenotype were not statistically significant [7]. These low-passage isolates were passed three times in Vero cells while our cells have a different passage history, especially BUNV which was passaged many times in suckling mouse brains while BATV and NRIV have relatively low passage histories. Given that our data also indicate the presence of small and large plaques based on the criteria devised by Odhiambo *et al*. [7], more research is needed to determine if passage history affects plaque makeup or if this is a consistent phenotype of these viruses even when freshly isolated from the field.

In all but one instance, the qRT-PCR primer/probe sets between shared gene segments cross-amplified (the NRIV M primers failed to cross-amplify BATV M). Using the NCBI BlastTool and the accession numbers from Table 1, we report that the BUNV and NRIV L segments have 7.37 % identity. When we compared the BATV and NRIV M segments, however, the percentage identity was reduced to 89.29 %. Thus, it is likely that sequencing is necessary to definitively identify M segments between these two viruses for the purposes of surveillance [40].

There were no significant differences in the growth kinetics – either magnitude of growth or DT – among the three viruses in Vero cell culture, and we established that these viruses retain infectiousness under laboratory conditions in acellular environments. Previous studies have reported *ex vivo* stability from other Bunyaviruses, particularly the survival of Hantaviruses in cell-free media [41, 42]. Other examples include Sandfly Fever Sicilian Virus (SFSV), a sandfly-borne virus; Crimean-Congo Haemorrhagic Fever Virus (CCHV), transmitted by ticks; and RVFV, which can be transmitted by mosquitoes [43–46]. The epidemiological relevance of extracellular stability is that it alters the route of transmission and risks associated with the handling of infected animals or contaminated waste. In the case of RVFV, environmental contamination from abortions or slaughter (and thus association with environmental stability) can be a major source of human-acquired cases [46]. Puumala orthohantavirus (PUUV) was shown to retain infectiousness extracellularly even under extreme conditions such as high temperature (56 °C) and desiccation [42]. However, this virus only retained infectiousness for 24 h at 37 °C, while BUNV, BATV and NRIV were still viable for 30 days extracellularly [42]. For these Orthobunyaviruses, it could be postulated that dead hosts and infective tissues might be sources of infectious viruses, provided permissive conditions (humidity, temperature, etc.).

At the laboratory level, this stability should be taken into account in the design and implementation of the laboratory safety measures by all staff manipulating these viruses. Importantly, we show that 1 % Triton-X-100 is effective in inactivating BUNV, BATV and NRIV. This could have useful applications such as for ELISA design as there are currently no such commercially available kits for these viruses. The use of inactivated viruses is especially important in field research and/or developing parts of the world that may lack appropriate biosafety facilities required to handle live viruses.

In summary, this study shows that NRIV is similar to its parental viruses BUNV and BATV in Vero cell culture. Whether this finding translates to *in vivo* studies in either putative vectors or vertebrate hosts should be further studied. In addition, these viruses are extracellularly stable, which has public health and biosafety implications. The study of this understudied group of viruses addresses a need for basic characterization of viruses that have not yet reached epidemic transmission intensity, but that have the potential due to their infectivity of both human and animal hosts [4, 19, 47].

## Supplementary Data

Supplementary material 1
