# Supplementary material for: Comparative characterization of the reassortant Orthobunyavirus Ngari with putative parental viruses, Bunyamwera and Batai: in vitro characterization and ex vivo stability
Source: J Gen Virol. 2020 Dec 1;102(2):001523. doi: 10.1099/jgv.0.001523 (PMC8116939; doi:10.1099/jgv.0.001523)

**Supplemental Information**

**Supplemental Figure S1: R**andomly chosen plaques (n=30) of BATV were measured and traced using ImageJ software (top). Original image (rendered black & white) on the bottom.

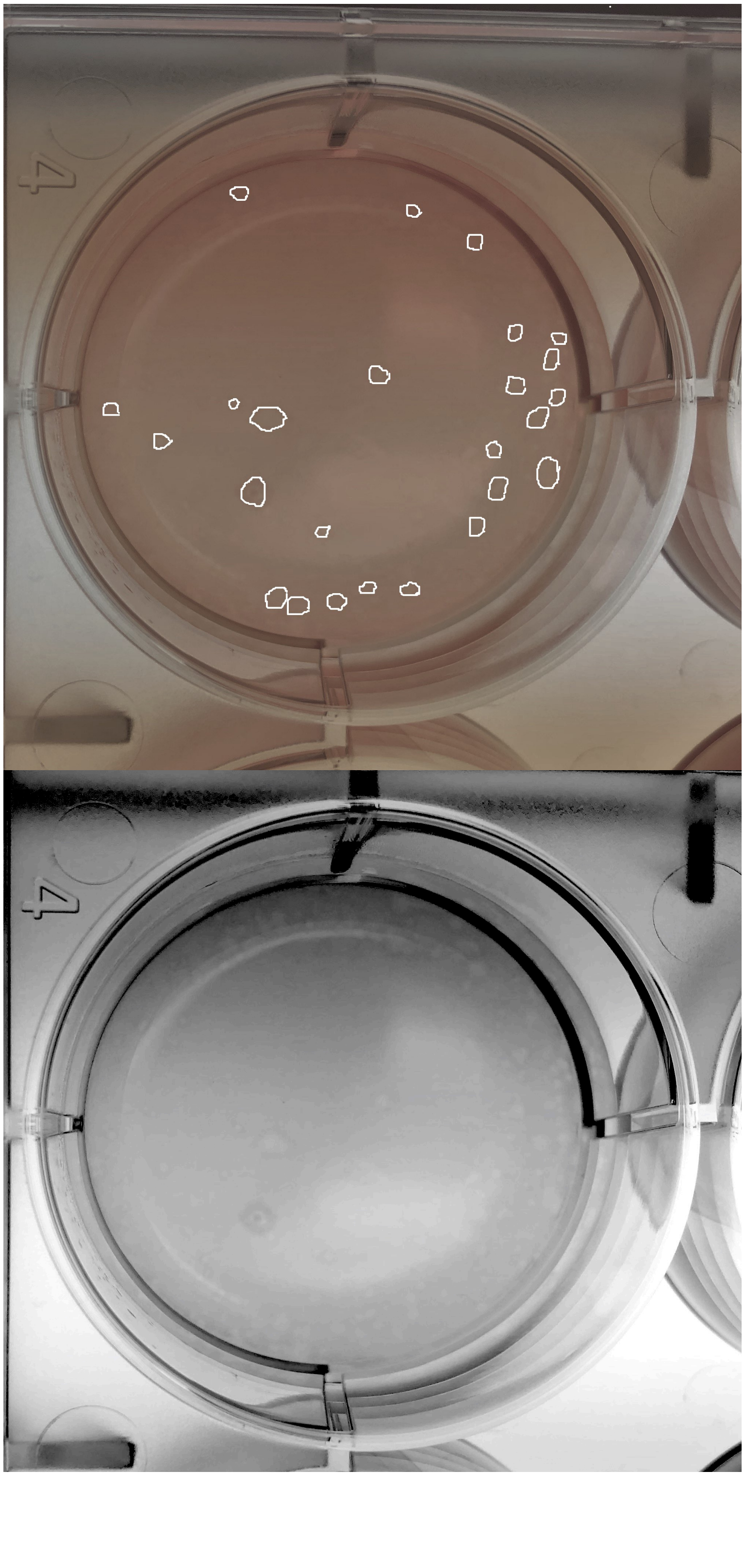

**Supplemental Figure S2:** Randomly chosen plaques (n=25) of BUNV were measured and traced using ImageJ software (top). Original image (rendered black & white) on the bottom.

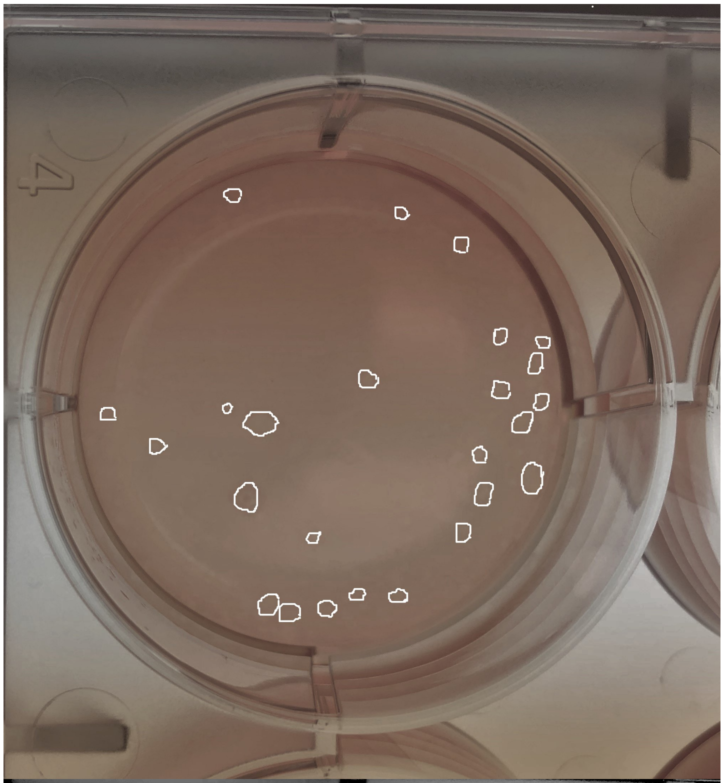


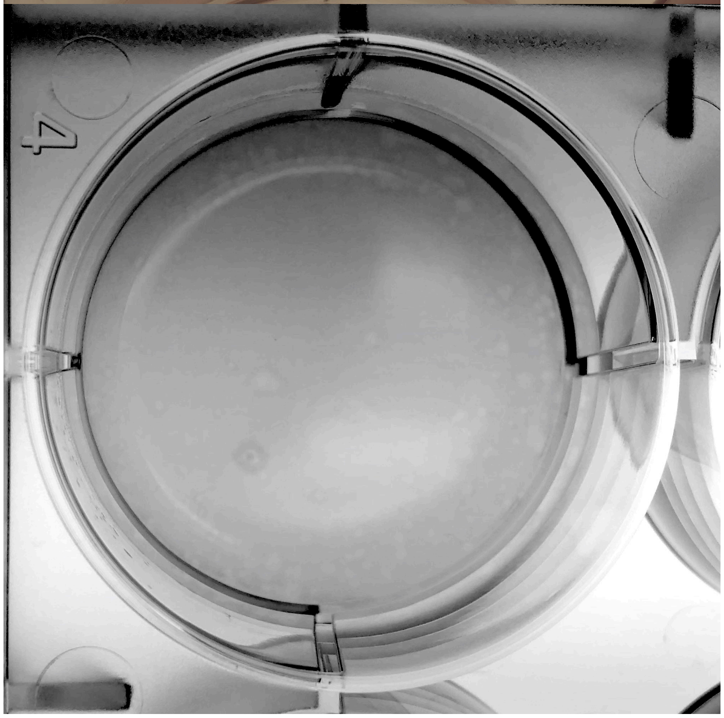

**Supplemental Figure S3: R**andomly chosen plaques (n=28) of NRIV were measured and traced using ImageJ software (top). Original image (rendered black & white) on the bottom

.

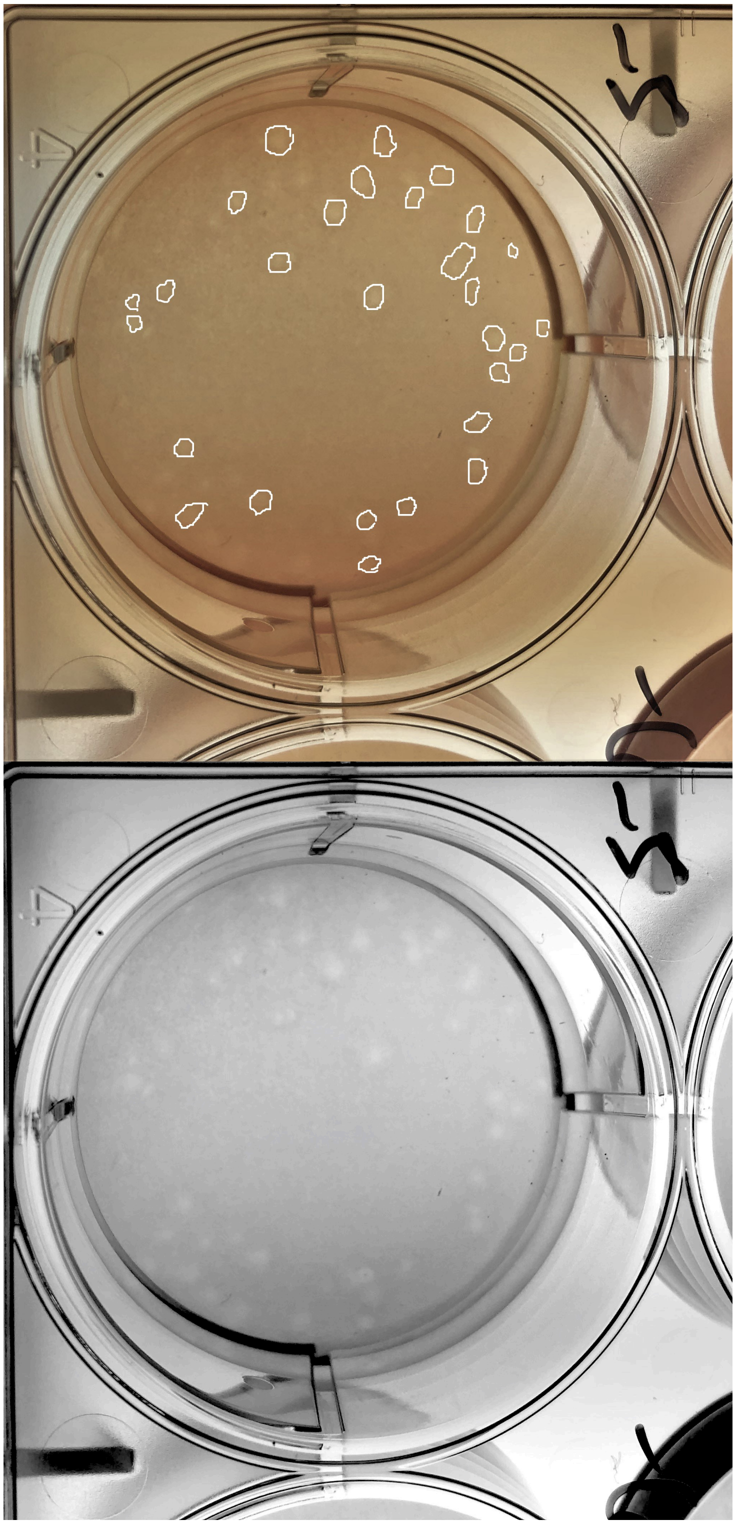

**Table S1**. **Homologous and cross-reactivity of qRT-PCR primers across the three viruses of interest**

| | | Homologous and Cross-reactivity | | |
|---|---|---|---|---|
| Primers/Probe | | BUNV | BATV | NRIV |
| BUNV | M | + | - | - |
| | L | + | - | + |
| BATV | M | - | + | + |
| | L | - | + | - |
| NRIV | M | - | - | + |
| | L | + | - | + |

**Supplemental Figure S4**: Plot of the Tukey HSD adjusted means and 95% confidence intervals for the AUC-L comparisons of initial inoculations doses of 1, 4, and 6 pfu/mL. All comparisons were not significant (p<.05), as is indicated by the non-overlap in the 95% CI to the null value of 0 difference (red line).

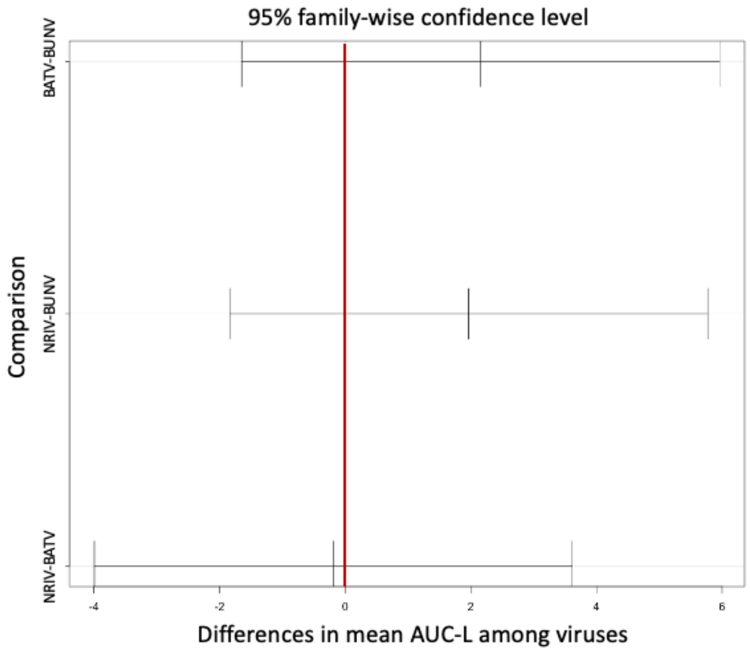

**Table S2:** Pairwise groupings from Tukey HSD of the main effect of initial inoculaton dose on the AUC_L.

| Effect | Initial inoculation titer (log pfu/mL) | Grouping according to TukeyHSD of initial inoculation titer |
|---|---|---|
| Initial Inoculation titer (Log10 PFU/mL ) | 6 | f |
| | 5 | d,e |
| | 4 | c,d |
| | 3 | a,b,c |
| | 2 | a,b |
| | 1 | a |

**Supplemental Figure S5**: Growth curves across all initial inoculation doses. Error bars represent daily standard deviations per virus.

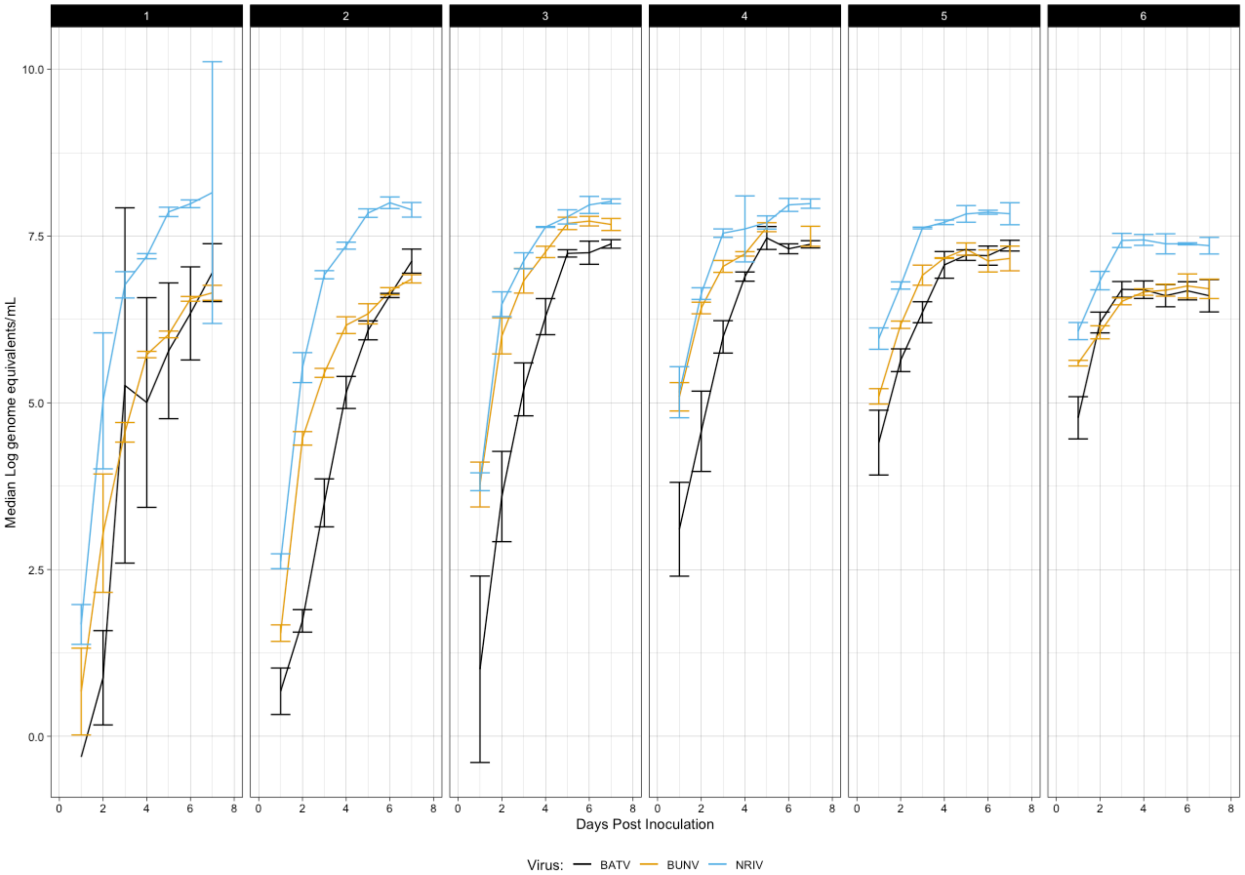

**Table S3:** Peak days and Doubling Times (DT) for growth curves of BUNV, BATV, and NRIV

| Virus | Initial inoculation titer (log pfu/mL) | Peak day | Doubling time (in days) |
|---|---|---|---|
| BUNV | 6 | 4 | 0.34 |
| | 5 | 5 | 0.25 |
| | 4 | 5 | 0.16 |
| | 3 | 5 | 0.37 |
| | 2 | 7 | 0.29 |
| | 1 | 7 | 0.38 |
| BATV | 6 | 4 | 0.10 |
| | 5 | 7 | 0.41 |
| | 4 | 5 | 0.45 |
| | 3 | 7 | 0.52 |
| | 2 | 7 | 0.51 |
| | 1 | 7 | 0.30 |
| NRIV | 6 | 4 | 0.10 |
| | 5 | 6 | 0.25 |
| | 4 | 6 | 0.41 |
| | 3 | 6 | 0.21 |
| | 2 | 6 | 0.27 |
| | 1 | 6 | 0.19 |

*TukeyHSD pairwise comparison was run based on an analysis of variance of the AUC-L on the main effect of initial inoculation titer.

**Table S4.** Viral RNA concentration of supernatants collected at 30 days post-inoculation on Vero cells.

| Virus | Replicate | Initial Inoculation Dose (Log/mL genome equivalents) | Genome equivalents (Log/mL) at 30 dpi |
|---|---|---|---|
| BATV | 1 | 6 | 6.152288 |
| BATV | 2 | 6 | 6.170262 |
| BATV | 3 | 6 | 6.328380 |
| BATV | 1 | 4 | 6.505150 |
| BATV | 2 | 4 | 6.604226 |
| BATV | 3 | 4 | 6.561101 |
| BATV | 1 | 1 | 6.830589 |
| BATV | 2 | 1 | 6.778151 |
| BATV | 3 | 1 | 6.589950 |
| BUNV | 1 | 6 | 5.348305 |
| BUNV | 2 | 6 | 5.530200 |
| BUNV | 3 | 6 | 5.401401 |
| BUNV | 1 | 4 | 6.456366 |
| BUNV | 2 | 4 | 6.515874 |
| BUNV | 3 | 4 | 6.178977 |
| BUNV | 1 | 1 | 6.494155 |
| BUNV | 2 | 1 | 6.600973 |
| BUNV | 3 | 1 | 6.790988 |
| NRIV | 1 | 6 | 7.123852 |
| NRIV | 2 | 6 | 7.247973 |
| NRIV | 3 | 6 | 7.146128 |
| NRIV | 1 | 4 | 7.659916 |
| NRIV | 2 | 4 | 7.630428 |
| NRIV | 3 | 4 | 7.627366 |
| NRIV | 1 | 1 | 7.462398 |
| NRIV | 2 | 1 | 7.576341 |
| NRIV | 3 | 1 | 7.650308 |

**Table S5:** Viral RNA concentration collected at 30 days post-inoculation into acellular media.

| Virus | Replicate | Genome equivalents (Log/mL) at 30 dpi |
|---|---|---|
| BATV | 1 | 4.846337 |
| BATV | 2 | 5.017033 |
| BATV | 3 | 5.100371 |
| BUNV | 1 | 4.680336 |
| BUNV | 2 | 4.912753 |
| BUNV | 3 | 4.842609 |
| NRIV | 1 | 5.973128 |
| NRIV | 2 | 5.858537 |
| NRIV | 3 | 5.841985 |

**Supplemental Figure S6:** Tukey HSD post-hoc comparisons of the AUC_L values within each virus type across Fresh (at 6 Log10/mL initial inoculation), 30 day-old cell culture inoculum, and 30-day old media inoculum.

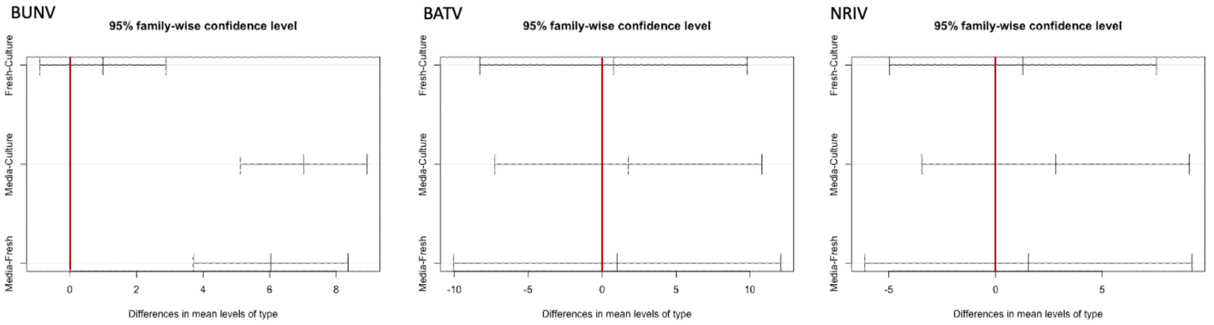

**Supplemental Figure S7:** No growth was observed from any of the viruses after inoculation of supernatant from cell culture treated with Triton-X 100, indicating successful inactivation of the viruses. Error bars are standard deviations from three replicates.

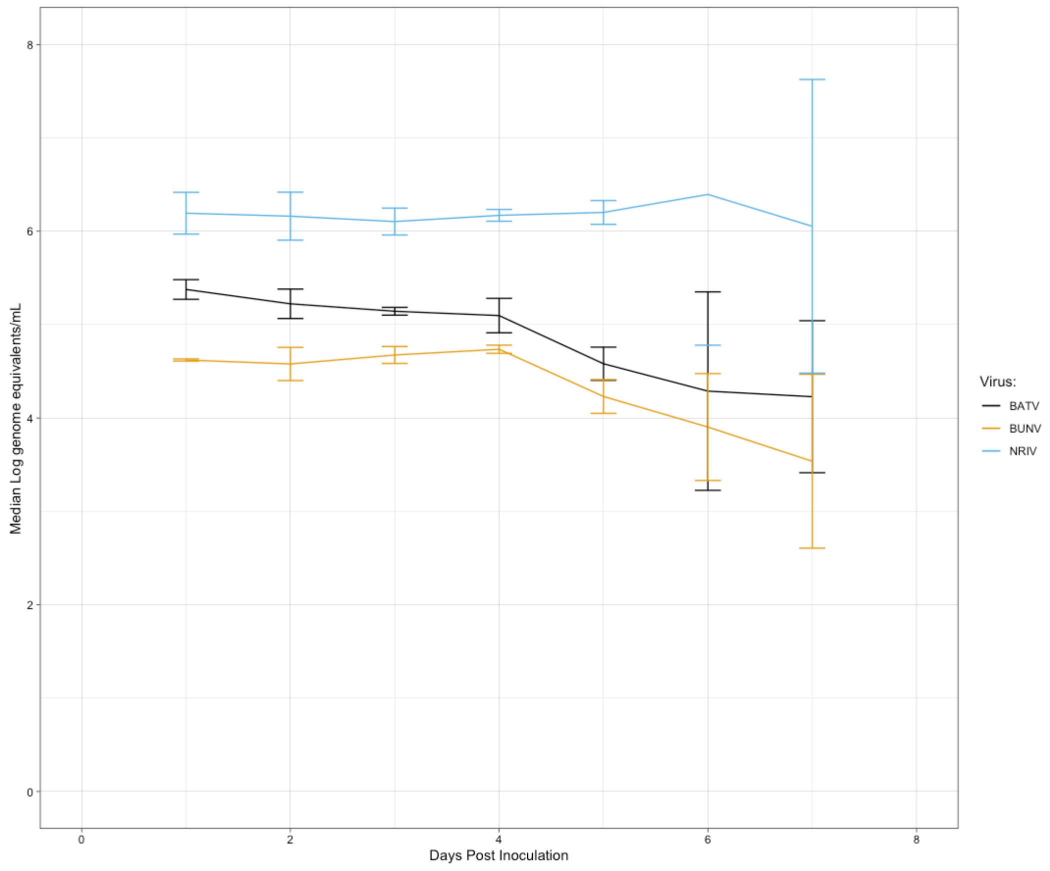

Supplement: Supplementary material 1 [file jgv-102-523-s001.pdf]
